# Supplementary material for: Multi-lens ultrasound arrays enable large scale three-dimensional micro-vascularization characterization over whole organs
Source: Nat Commun. 2025 Oct 28;16:9317. doi: 10.1038/s41467-025-64911-z (PMC12569009; doi:10.1038/s41467-025-64911-z)
Supplement: Supplementary file 1 — Supplementary Information [file 41467_2025_64911_MOESM1_ESM.pdf]

Multi-lens ultrasound arrays enable large scale three-dimensional micro-vascularization characterization over whole organs

Supplementary Figures

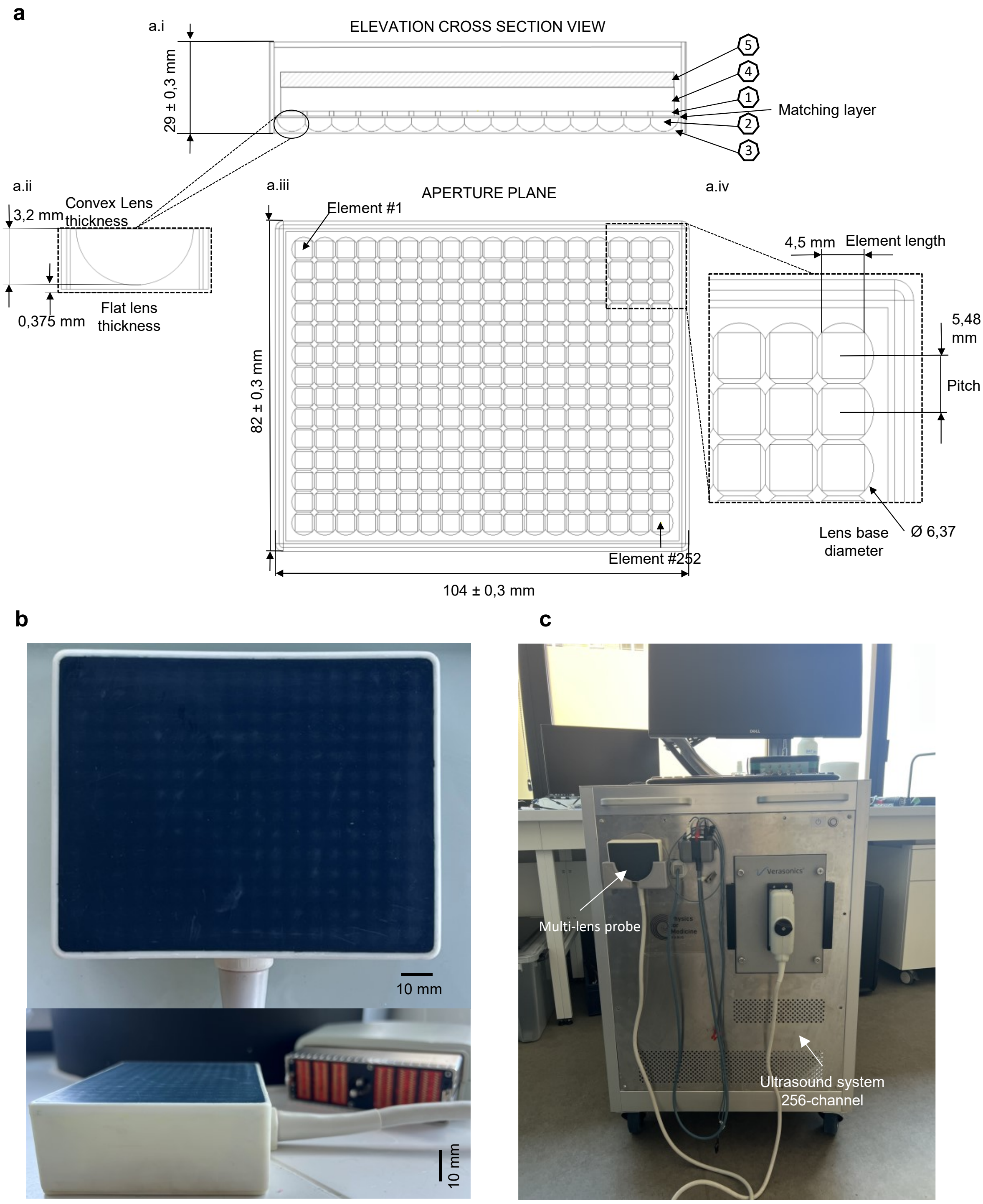

Supplementary Fig. 1|Technical schematics and photograph of the custom 252-element multi-lens probe and ultrasound imaging system. a, Transducer specifications and dimensions: (**a.i**) Elevation cross section view of the probe showing the layers containing; **1** represents the composite of piezoelectric elements, **2** represents the convex lens matrix layer, **3** flat lens layer which is the silicon concave lens, **4** backing layer, **5** mechanical stiffener layer. (**a.ii**) Magnified view of a single compound lens shown the convex lens with a thickness of 3.2 mm and the concave lens on top with a maximum thickness of 0.375 mm. (**a.iii**) View of the probe's aperture plane, illustrating the total aperture size (104 mm × 82 mm) and the number of transducer elements (252). (**a.iv**) Magnified view of the probe's aperture plane, showing the geometry of a single transducer elements (side length: 4.5 mm), pitch (5.48 mm), and lens base diameter (6.37 mm). (**a.iv**) Magnified view of the probe's aperture plane, showing the geometry of a single transducer elements (side length: 4.5 mm), pitch (5.48 mm), and lens base diameter (6.37 mm). Panels created using Autodesk Inventor Professional 2025.0.1.**b,** Photograph of the custom 252-element multi-lens probe, showing the aperture plane at the top and the elevation view at the bottom of the image. **c,** Photograph of the multi-lens probe connected to the 256-channel ultrasound imaging system.

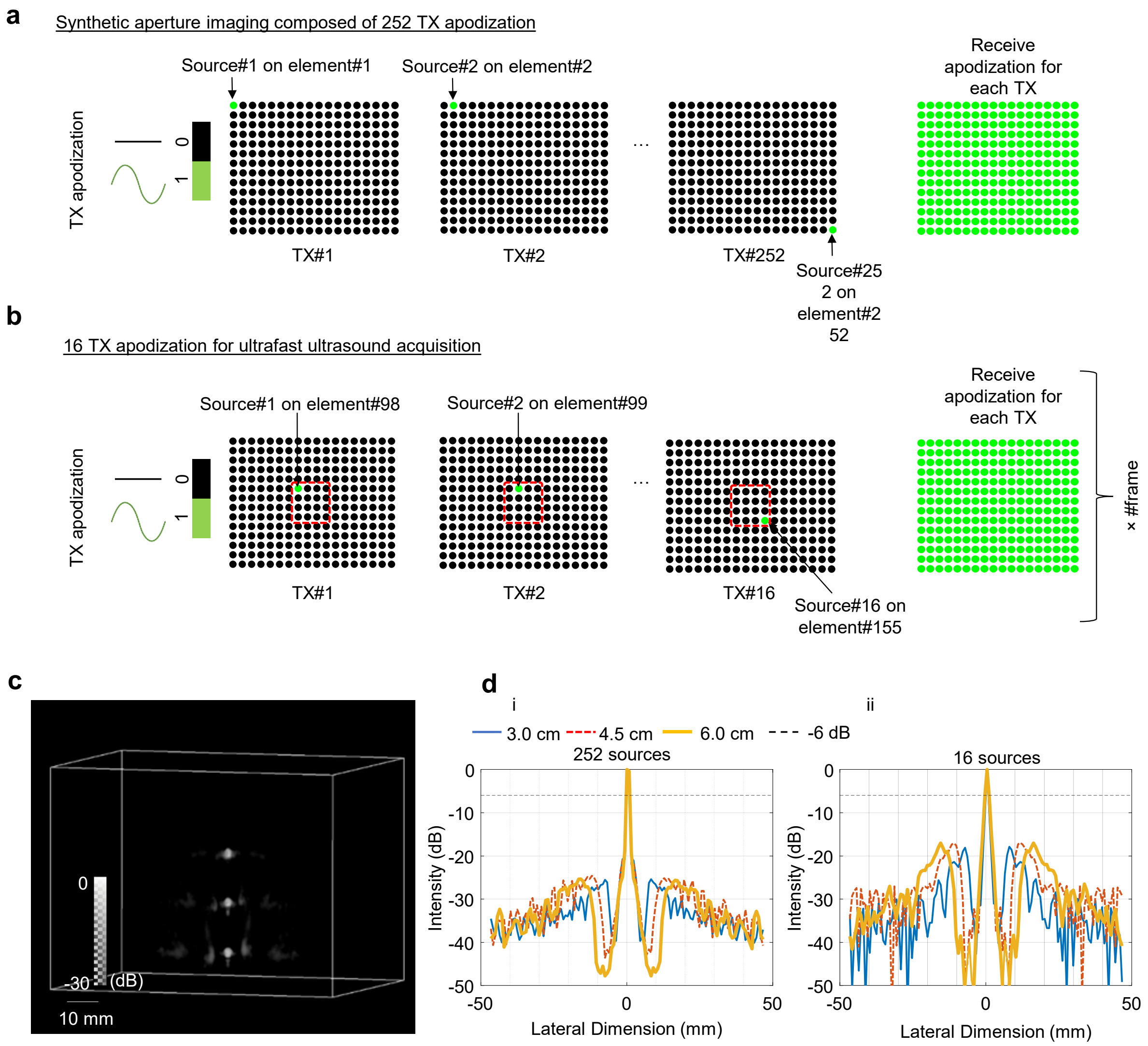

Supplementary Fig. 2| Ultrasound imaging sequences and simulation validation of the point spread function with the Multi-Lens Probe. a, Schematic illustration of synthetic aperture imaging (SAI) using the multi-lens probe, showing 252 transmit apodization (TX) where each source is activated individually (active element: green dot, non-active element: black dot) on a single element during each transmission. During reception, all elements are activated after every transmit event TX. **b**, Schematic illustration of the ultrafast acquisition scheme using the multi-lens probe, showing 16 transmit apodizations (TX), where each source is activated individually (green dot) on a single element during each transmission. During reception, all elements are activated after every transmit event. **c**, Numerical 3D point spread functions (PSFs) of the multi-lens array using SAI, evaluated at the center of the medium for depths of 3 cm, 4.5 cm, and 6 cm. **d**, Quantitative comparison of the PSF intensity profiles at –6 dB for SAI (panel **d.i**) and ultrafast transmission with 16 sources (panel **d.ii**). The blue graph represents the maximum intensity projection profile of the PSF along the lateral dimension at a depth of 3 cm, while the dashed red and solid yellow graphs correspond to depths of 4.5 cm and 6 cm, respectively.

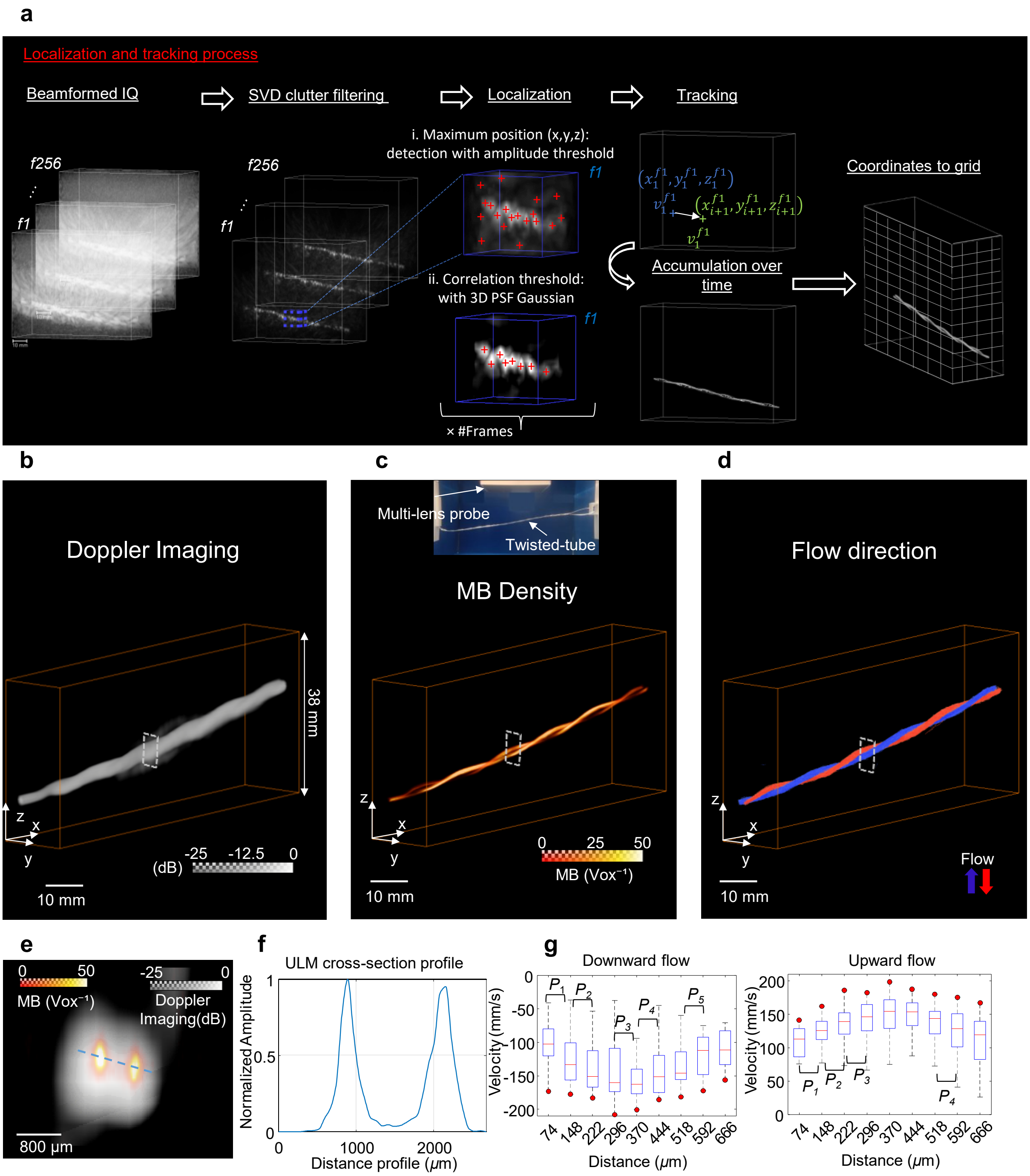

Supplementary Fig. 3| Localization and tracking process pipeline and validation on twisted tube phantom. a, Schematic illustration of the ULM processing pipeline, starting from IQ beamformed data, followed by SVD-based clutter filtering, microbubble (MB) localization, MB tracking, and final 3D rendering of the phantom. **b**, Volumetric enhanced-contrast doppler of the twisted tube. **c,** 3D MB density of the twisted tube for an imaging volume of (38 × 93 × 16 mm³). Top of (**c**): experimental setup photograph image showing the multi-lens probe and twisted tube in a water tank. **d**, 3D directional flow velocity map, blue indicates upward flow while red represents downward flow. arrows indicate upward (dark blue) and downward (dark red) flow directions. **e**, Cross-section of the twisted tube (white box in **c**) from the ULM image, overlaid with enhanced-contrast Doppler imaging (white box in **b**), highlighting the separation between the intertwined tube segments as resolved by ULM. **f**, Distance profile (blue curve) across the cross-section of the twisted tube shown in panel (**e**). **g**, Cross-sectional velocities in the twisted tube reveal a characteristic Poiseuille flow profile, with downward flow on the left and upward flow on the right of panel (**g**). Significant differences between the neighbouring voxels (~75 µm) within the velocity profile were demonstrated using an unpaired two-sided Student's t-test. P values for downward flow from left to right are $P_1 = 0.0477$, $P_2 = 6.89 \times 10^{-05}$, $P_3 = 0.0003$, $P_4 = 0.0005$, $P_5 = 0.0108$. P values for upward flow from left to right are $P_1 = 3.99 \times 10^{-09}$, $P_2 = 0.0077$, $P_3 = 0.0475$, $P_4 = 7.82 \times 10^{-09}$. The red horizontal line indicates the median; boxes denote the 25th and 75th percentiles; whiskers extend to the most extreme data points not considered outliers. n samples correspond to the number of microbubbles (MBs) passing through each voxel (63, 94, 192, 210, 234, 224, 127, and 57 from left to right for downward flow; 35, 130, 228, 454, 568, 459, 337, and 174 from left to right for upward flow). other points, outliers. Measurements are technical replicates.

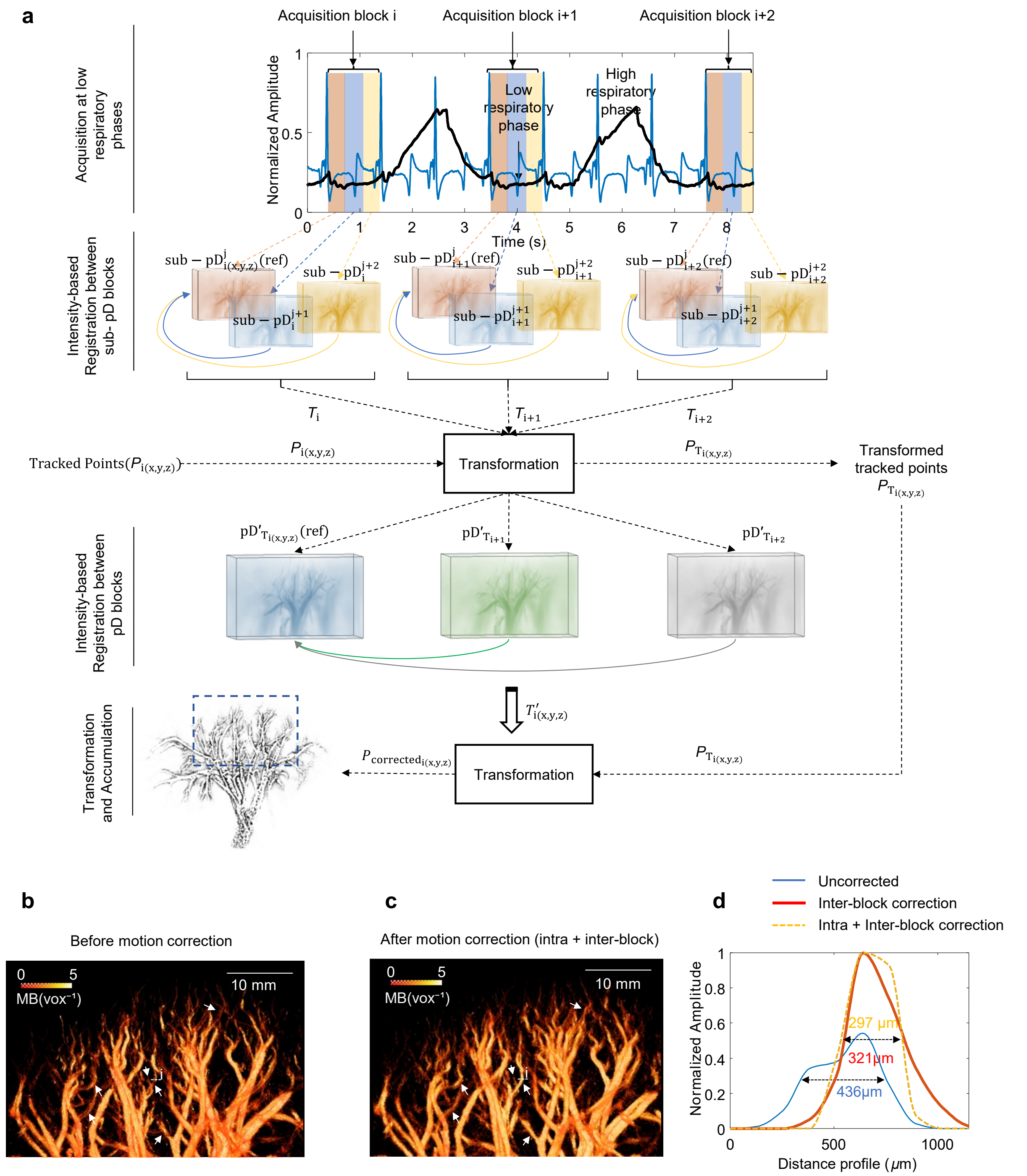

Supplementary Fig. 4| Intra-block motion correction pipeline using 3D power Doppler intensity rigid registration. **a**, Illustration of acquisition blocks obtained during low respiratory phases and synchronized with the cardiac cycle. Each acquisition is divided into three sub-phases, and the corresponding power Doppler volumes are reconstructed and color-coded by phase. $\mathrm{Sub-pD}^{j}_{i(x,y,z)}$ denotes the sub power doppler block used as the reference for 3D intensity-based rigid registration for the others sub power dopplers within one block. $T_{i_{(x,y,z)}}$ represents the transformation parameters resulting from registration between the reference sub-volume and each sub-volume to be aligned. $P_{i_{(x,y,z)}}$ corresponds to the microbubble (MB) tracked positions within each sub-block prior to correction, and $P_{T_{i(x,y,z)}}$ represents the MB positions after intra-block correction. The concatenated result of all sub-volumes after rigid transformation is denoted as $\mathrm{pD'}_{T_{i(x,y,z)}}$ which constitutes the full power Doppler volume for one cardiac cycle. At the inter-block level, $T'_{i_{(x,y,z)}}$ denotes the transformation parameters derived from intensity-based rigid registration between the reference power Doppler block and each subsequent block. $P_{\mathrm{corrected}_{i(x,y,z)}}$ represents the MB positions within each block after inter-block motion correction. **b**, Density map of the kidney cortex (blue box; ~ 28 × 45 × 33 mm³) before motion correction. **c**, Density map of the kidney cortex (blue box; ~ 28 × 45 × 33 mm³) after intra-block motion correction. **d**, Comparison of Cross-sectional density profile of vessel (i) in both panel b and c, showing the normalized amplitude as a function of distance before (blue curve), inter-block (solid red curve), and intra-block (yellow dashed curve) motion correction.

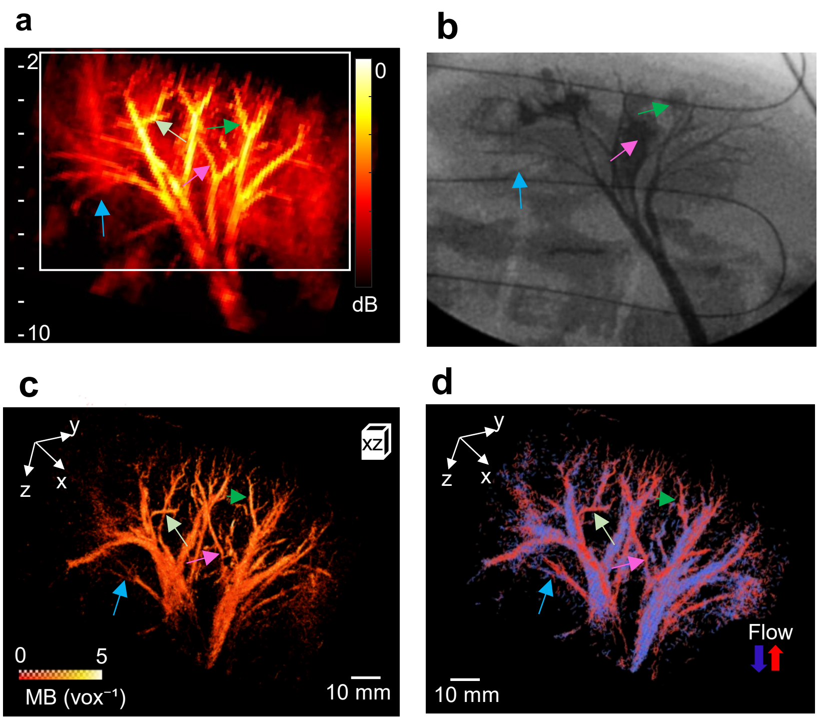

Supplementary Fig. 5| In vivo kidney of porcine 1. a, Maximum Intensity Projection in the XZ plane of contrast-enhanced Power Doppler imaging from a porcine kidney, acquired using the custom multilens array probe; the arrows show the similarity of the trees between the panel **a** and **b**. **b**, Renal artery angiography of the porcine kidney. **c,** 3D MB density map of the porcine kidney showing the size of imaging volume (60 x 90 x 30 mm^3) **d**, Directional flow velocity 3D map that identifies the arterial (red) and venous (blue) flow. arrows indicate upward (dark red) and downward (dark blue) flow directions.

Supplementary Methods

Principles and definitions. A multi-lens array is a matrix array of large transducer elements combined with diverging lenses. A transducer element is considered large if its sides are larger than three times the acoustic wavelength. Large elements enable to reduce the number of elements required to fulfil a large aperture probe, overcoming the technological complexities associated with fully populated matrix arrays. Due to their large individual area in receive, the sensitivity is also enhanced. Large elements are naturally highly directive and diverging lenses placed in front of them enable to decrease individual element directivity and therefore increase the antenna gain.

An acoustic lens is governed by Snell's law:

$$\sin\theta_1 \times c_2 = \sin\theta_2 \times c_1 \qquad \text{Supplementary Equation (1)}$$

where θ_1 and θ_2 are the incident and refractive angles, respectively. c_1 and c_2 are the speed of sound in the incident and refractive media, respectively. The element surface being plane, the lens can be plano-convex or plano-concave. To make a plano-convex diverging lens the incidence angle must be larger than the refractive angle which implies:

$$\frac{\sin\theta_1}{\sin\theta_2} < 1 \Rightarrow c_1 > c_2 \qquad \text{Supplementary Equation (2)}$$

Similarly, to make a plano-concave diverging lens the incidence angle must be smaller than the refractive angle which implies:

$$\frac{\sin\theta_1}{\sin\theta_2} > 1 \Rightarrow c_2 > c_1 \qquad \text{Supplementary Equation (3)}$$

A compound lens[1] can be made of a plano-convex lens with a speed of sound of c_1 combined to a plano-concave lens with a speed of sound of c_2. The final refractive medium being the biological soft tissues c_3.Thus, to make a compound diverging lens:

$$c_1 > c_2 \text{ and } c_3 > c_2$$

According to Supplementary Equation (2) and Supplementary Equation (3)

To maximize energy transmission from the transducer to biological soft tissues, an impedance matching layer between the transducer and the lens must be considered. The acoustic impedance of the compound lens should be appropriately chosen to lie between that of the transducer and that of the tissue. Therefore, the density of the compound lens is a key parameter, as it directly influences its acoustic impedance.

Beamforming processing. Coherent compounding and dynamic focusing were performed. A 3D delay-and-sum beamforming was achieved with a lens delay correction to take into account the acoustic travel path through the compound lens[2]. 4D IQ volumes (In-phase and Quadrature) were computed with a pitch grid of λ/2 in each spatial directions (x, y, z) to obtain one block of beamformed volumes. Process was repeated for each block. Processing was performed using MATLAB software (2021a, The MathWorks Inc., USA). The beamforming process was implemented in CUDA language and processed on GPU units.

3D ultrasound localization microscopy data processing. 4D IQ volumes were filtered using the spatio-temporal SVD filter [3]. The SVD filter was applied for each block by removing the first eigenvectors (between 12 and 55 of the first eigenvectors depending on the level of the tissue) to remove tissue signals and keep only the echoes coming from moving MBs, resulting in so-called filtered 4D IQ volumes. Next, 3D Power Doppler volumes were generated by integrating the filtered IQ volumes over time and saved for each acquisition block to be used for 3D motion correction.

Filtered 4D IQ volumes were then processed for microbubbles localization and tracking using the same algorithm described in [4,5]. Microbubble identification was performed by detecting local maxima that were cross-correlated with a Gaussian point spread function (PSF) previously characterized from in vitro measurements. Local maxima were retained as microbubbles if their intensity was above the 99th percentile of the absolute values of the filtered IQ volume (Supplementary Fig. 3.a.i) and their cross-correlation coefficient exceeded a threshold of 0.6

(Supplementary Fig. 3.a.ii). These dual thresholds effectively suppressed grating lobes by leveraging their lower intensity and distinct shape compared to the main lobe corresponding to the microbubble. MB center positions were stored over time to be tracked. For the tracking, the Simpletracker algorithm was used, which is based on the Hungarian algorithm (https://github.com/tinevez/simpletracker, Jean-Yves Tinevez, 2021). A maximum linking distance of 0.96 mm (approximately 2 pixels) between two subsequent positions was allowed. Tracks smaller than 5 frames were rejected. The tracks were used to compute velocity measurements in three dimensions as explained in Heiles et al [6]. The MB density maps were reconstructed based on the center position of the microbubbles. By specifying a voxel size, the intensity value was determined by the number of detected MB within that voxel. In a similar manner, velocity maps were reconstructed based on the velocity magnitude of each MB or one of their velocity components along the x, y, or z axis. This led to the creation of quantitative and localized maps showing the velocity of blood flow in the organ's vasculature.

3D dynamic ULM and resistivity index. After applying the tracking algorithm to all acquisition blocks, frame selection was performed. The selection began with the first frame corresponding to the R-peak in the ECG and ended with the frame marking the end of the systolic phase. The end of the systolic phase was determined by calculating the average of the systolic endpoints across all blocks according to their ECG signal. Subsequently, a mean of 20 frames was computed over the systolic phase. For the diastolic phase, a mean of 20 frames was also applied to the remaining frames, as each acquisition block concludes at the end of the cardiac cycle. The velocities during the two phases were quantified by measuring the mean velocity within selected vessels [7,8].

The Resistivity Index (RI) was calculated using Supplementary Equation (4) below, which V_{sys} represents the peak systolic velocity, and V_{end} denotes the end-diastolic velocity

$$\text{RI} = \frac{V_{\text{sys}} - V_{\text{end}}}{V_{\text{sys}}} \qquad \text{Supplementary Equation (4)}$$

3D visualization. For *ex vivo* porcine heart and *in vivo* porcine kidney results, the 3D MB density and velocity maps were reconstructed using isotropic voxel sizes of $\lambda/10$, based on all tracked MB coordinates. For the *in vivo* porcine liver results, voxel sizes of $\lambda/8$ were applied. Meanwhile, for the *in vitro* experiments of the tube, isotropic voxel sizes of $\lambda/20$ were used. The reconstructed volumes were then saved and exported as Niftii files. These files were later opened, and vessel representations were rendered using the volume rendering (volren) function of Amira software (Amira 3D 2021.1, Visualization Sciences Group, USA).

3D flow and resolution quantifications. An orthogonal slice of the tube velocity map of $\lambda/4$ thickness was assessed. The velocities tracks along the thickness dimension were kept to perform statistical student's t-test between the neighbouring voxels along the velocity profile (voxel size = 75 µm). A p value less than 0.05 was considered statistically significant. This quantification aims to extract the spatial resolution for *in vitro* study and to determine the velocity profile.

The flow rate assessment through the tube as a function of the inlet flow rate during the experiment was determined by measuring the inner diameter of the tube and the maximum velocity at the center of the tube, assuming a Poiseuille flow:

$$Q = \frac{V_{\text{max}}}{2}\pi R^2 \qquad \text{Supplementary Equation (5)}$$

Where Q is the flow rate, V_{max} is the maximum velocity, and R is the radius.

Vessel's diameters in *ex vivo* and *in vivo* results were estimated using skeletonization based on the following process: from the 3D density map reconstructed with isotropic voxels of 150 µm in Amira software, a Gaussian filter was applied, followed by the 'auto-skeleton' function, which generated a skeleton in regions where the density value exceeded 1. The radius was then computed for each centerline point in all detected vessels.

The estimated spatial resolution of 3D coronary mapping of the isolated porcine heart and the vasculature of the *in vivo* porcine kidney was performed using the Fourier Shell Correlation (FSC)[9]. The calculation was achieved by adapting code from (https://github.com/bionanoimaging/cellSTORM-MATLAB). The intersection between the FSC with half-bit threshold curve was used to determine the resolution. A voxel size of $\frac{1}{30} \times \frac{1}{30} \times \frac{1}{30}\lambda^3$ was used for an adequate spatial frequency range.

The flow rate as function of the radius was determined through the following steps: after the reconstruction of the MB density map, it was binarized using 3D Jerman vesselness enhancement filter, in order to cancel the background noise [10]. The centerline points were derived from this mask, and the radius was approximated at every point along the resulting centerline. This process was performed using the open-source software VesselVio [11]. The centerline velocity with the radius estimation has been included to calculate the average flow rate at each centerline point, assuming a Poiseuille flow. The flow rate was traced as function of the estimated radius to fit a power law.

Supplementary References

1. Yang, S. *et al.* Design and evaluation of a compound acoustic lens for photoacoustic computed tomography. *Biomed. Opt. Express, BOE* **8**, 2756–2765 (2017).
2. Favre, H., Pernot, M., Tanter, M. & Papadacci, C. Boosting transducer matrix sensitivity for 3D large field ultrasound localization microscopy using a multi-lens diffracting layer: a simulation study. *Phys. Med. Biol.* **67**, 085009 (2022).
3. Demené, C. *et al.* Spatiotemporal Clutter Filtering of Ultrafast Ultrasound Data Highly Increases Doppler and fUltrasound Sensitivity. *IEEE Trans Med Imaging* **34**, 2271–2285 (2015).
4. Demeulenaere, O. *et al.* In vivo whole brain microvascular imaging in mice using transcranial 3D Ultrasound Localization Microscopy. *EBioMedicine* **79**, 103995 (2022).
5. Demeulenaere, O. *et al.* Coronary Flow Assessment Using 3-Dimensional Ultrafast Ultrasound Localization Microscopy. *JACC Cardiovasc Imaging* **15**, 1193–1208 (2022).
6. Heiles, B. *et al.* Ultrafast 3D Ultrasound Localization Microscopy Using a 32 × 32 Matrix Array. *IEEE Trans Med Imaging* **38**, 2005–2015 (2019).
7. Bourquin, C. *et al.* Quantitative pulsatility measurements using 3D dynamic ultrasound localization microscopy. *Phys. Med. Biol.* **69**, 045017 (2024).
8. Bourquin, C., Porée, J., Lesage, F. & Provost, J. In Vivo Pulsatility Measurement of Cerebral Microcirculation in Rodents Using Dynamic Ultrasound Localization Microscopy. *IEEE Transactions on Medical Imaging* **41**, 782–792 (2022).
9. Heiles, B. *et al.* Volumetric Ultrasound Localization Microscopy of the Whole Rat Brain Microvasculature. *IEEE Open Journal of Ultrasonics, Ferroelectrics, and Frequency Control* **2**, 261–282 (2022).
10. Jerman, T., Pernuš, F., Likar, B. & Špiclin, Ž. Beyond Frangi: an improved multiscale vesselness filter. in *Medical Imaging 2015: Image Processing* vol. 9413 623–633 (SPIE, 2015).
11. Bumgarner, J. R. & Nelson, R. J. Open-source analysis and visualization of segmented vasculature datasets with VesselVio. *Cell Reports Methods* **2**, 100189 (2022).
